# Predicting resolution of hypoglycemia with and without dextrose infusion in newborn infant of gestational diabetic mothers

**DOI:** 10.3389/fped.2022.1039219

**Published:** 2022-11-30

**Authors:** Mohammed Ibrahim, Wei Hou, Joseph Decristofaro, Echezona T. Maduekwe

**Affiliations:** ^1^ Department of Pediatrics, Neonatology, Stony Brook Children's Hospital, Stony Brook, New York, United States; ^2^ Family, Population and Preventive Medicine, Stony Brook University Hospital, Stony Brook, New York, United States

**Keywords:** neonatal hypoglycemia, infant of gestational diabetic mothers, intravenous dextrose infusion, score system, term infants

## Abstract

**Introduction:**

Neonatal hypoglycemia (NH) may lead to significant neurological impairment if left untreated. Infants of gestational diabetic mothers (IGDM) are at increased risk of early NH and need to be screened. However, it is challenging to predict management with or without intravenous dextrose once hypoglycemia is identified. We evaluated the association between hypoglycemia risk scores at 1-hour of life and the need for intravenous dextrose for hypoglycemia resolution in IGDM.

**Methods:**

This was a retrospective cohort study of IGDM born at a gestational age ≥35 weeks from January 2015 to December 2017. NH was the disease of interest. The outcomes were the association of hypoglycemia risk score (HRS) with and without intravenous dextrose for hypoglycemia resolution. Each infant's hypoglycemia risk score (HRS) was calculated using data extracted from the maternal and neonatal electronic medical records. Resolution of hypoglycemia with and without intravenous dextrose was compared between the low HRS (0–1) group and the high HRS (2–5) group.

**Results:**

Sixty-five infants were included in the study with a mean gestational age of 38.2 ± 1 weeks for low HRS and 38.0 ± 2 weeks for high HRS. While more children with high HRS were delivered by cesarean section (*p* = 0.04), hypoglycemia resolved more frequently without intravenous dextrose in infants with low HRS (*p* = 0.03).

**Conclusion:**

IGDM is at increased risk of NH. The resolution of hypoglycemia without dextrose infusion is frequently associated with low HRS at 1-hour of life. Early identification using HRS of IGDM whose hypoglycemia will resolve with or without intravenous dextrose may help clinicians triage newborns to either stay in the nursery or transfer for more invasive care.

## Introduction

NH is a common diagnosis in newborns, and infants of diabetic mothers are at higher risk than infants of nondiabetic mothers. It occurs in 15%–25% of IGDM despite advancements in the perinatal management of diabetic mothers (1). This increased risk of NH in the IGDM is severe in infants of mothers with poorly controlled diabetes due to the relative fetal hyperinsulinemia developed in response to high maternal glucose levels (2, 3). The glucose level of 47 mg/dl (2.6 mmol/L) is usually considered a clinical threshold for the definition of hypoglycemia because levels <47 mg/dl (2.6 mmol/L) trigger concern for permanent neurological sequelae (4–7). This safety concern for infants at risk for NH led to the generation of guidelines by the American Academy of Pediatrics (AAP) to manage NH.

The AAP focuses on at-risk infants or infants with symptomatic hypoglycemia in the first 24 h of life. For all asymptomatic IGDM, AAP recommends starting glucose screening within an hour of life. Glucose levels <25 mg/dl (1.4 mmol/L) within 4 h of age despite initiating enteral feed indicate the need for intravenous dextrose. After the first 4 h of life, the hourly screening goal is to maintain pre-prandial glucose levels >45 mg/dl (2.5 mmol/L) for up to 12 h. While glucose levels <35 mg/dl (1.9 mmol/L) indicate the need for intravenous dextrose, borderline levels between 35 and 45 mg/dl (1.9–2.5 mmol/L) trigger continuing glucose monitoring (8). These continuous monitoring results from the inability to predict which infants will still have NH that will require intravenous dextrose for resolution.

Given the need for recurrent glucose monitoring in IGDM until 12 h of age, we aimed to evaluate the association between the HRS (9) at an hour of life in IGDM born at ≥35 weeks gestational age and the resolution of NH with or without intravenous dextrose.

## Materials and methods

A retrospective chart review was undertaken of all hypoglycemic IGDMs born at ≥35 weeks gestational age in our institution for 2 years (January 2015 and December 2017). We excluded infants with conditions that affected glucose levels like prenatal exposure to beta-agonists, known metabolic disorders, or congenital malformations.

As part of our routine care, newborn IGDM were admitted to the NBN if born at ≥36 weeks' gestation, tolerated oral feed, and had a glucose level of ≥25 mg/dl (1.4 mmol/L) within an hour of life. All neonates <36 weeks' gestation or those with glucose levels <25 mg/dl (1.4 mmol/L) were admitted to the NICU. All POC glucose was done at the bedside with the Stat strip® glucometer (Nova Biomedical, Waltham, MA, USA). POC glucose <2.2 mmol/L was redrawn from plasma glucose and measured in the central biochemistry laboratory with a Hexokinase-mediated reaction Roche Cobas C501 chemistry analyzer.

Furthermore, as part of our institution routine in the NICU, the growth size of all patients enrolled in this study was evaluated with the Fenton growth curve.

We used the plasma glucose level <47 mg/dl (2.6 mmol/L) as the operational definition of hypoglycemia (10, 11). We followed the AAP guidelines (8) for post-delivery glucose screening of IGDM during the study period. None of the subjects in this study received glucose gel, as it had not become the standard of care in our institution at the time. All POC glucose levels <2.2 mmol/L were redrawn within 1–2 min of obtaining the POC glucose for laboratory confirmation with plasma glucose.

The following information was extracted from the maternal hospital's electronic medical records: age, delivery method (vaginal or cesarean), body mass index (BMI), and pre-delivery glucose. We calculated the HRS for all the subjects at an hour of life using maternal age, maternal pre-delivery glucose, neonatal weight for age, and neonatal blood glucose levels. We considered a HRS of 0–1 as low and a score ≥2 as high, as shown in Table 1.

**Table 1:** Hypoglycemia risk score (HRS).

Independent Factors	0 point	1 point	2 points
Maternal Age	<35 years	≥35 years	n/a
Maternal pre-delivery glucose level (mmol/L)	<6.66	≥6.66	n/a
Neonatal weight for age	AGA	SGA, LGA	n/a
Neonatal glucose (mmol/L)	≥2.22 or <6.66	n/a	<2.22 or ≥6.66

Hypoglycemia Risk Score (HRS) components. AGA, appropriate for gestational age; SGA, small for gestational age; LGA, large for gestational age.

A power analysis determined that a sample size of 65 IGDM was necessary to identify infants whose hypoglycemia would resolve without intravenous dextrose with a sensitivity of 95% and a specificity of ≥80%. We used the Chi-square and t-test to compare the demographic variables between infants with HRS 0–1 vs. HRS 2–5. A multiple logistic regression model was used to evaluate the relationship between the HRS (0–1 vs. 2–5) and the gestational age, gender, birth weight, mode of delivery, maternal age, maternal pre-delivery glucose, and maternal BMI.

This research was approved by the SUNY Stony Brook Institution Review Board approved the study. The data were analyzed using the SAS v9.4 (SAS Institute, Cary, NC) for statistical analysis, and we considered differences at *p* values <0.05 as statistically significant.

## Results

**Figure 1:**
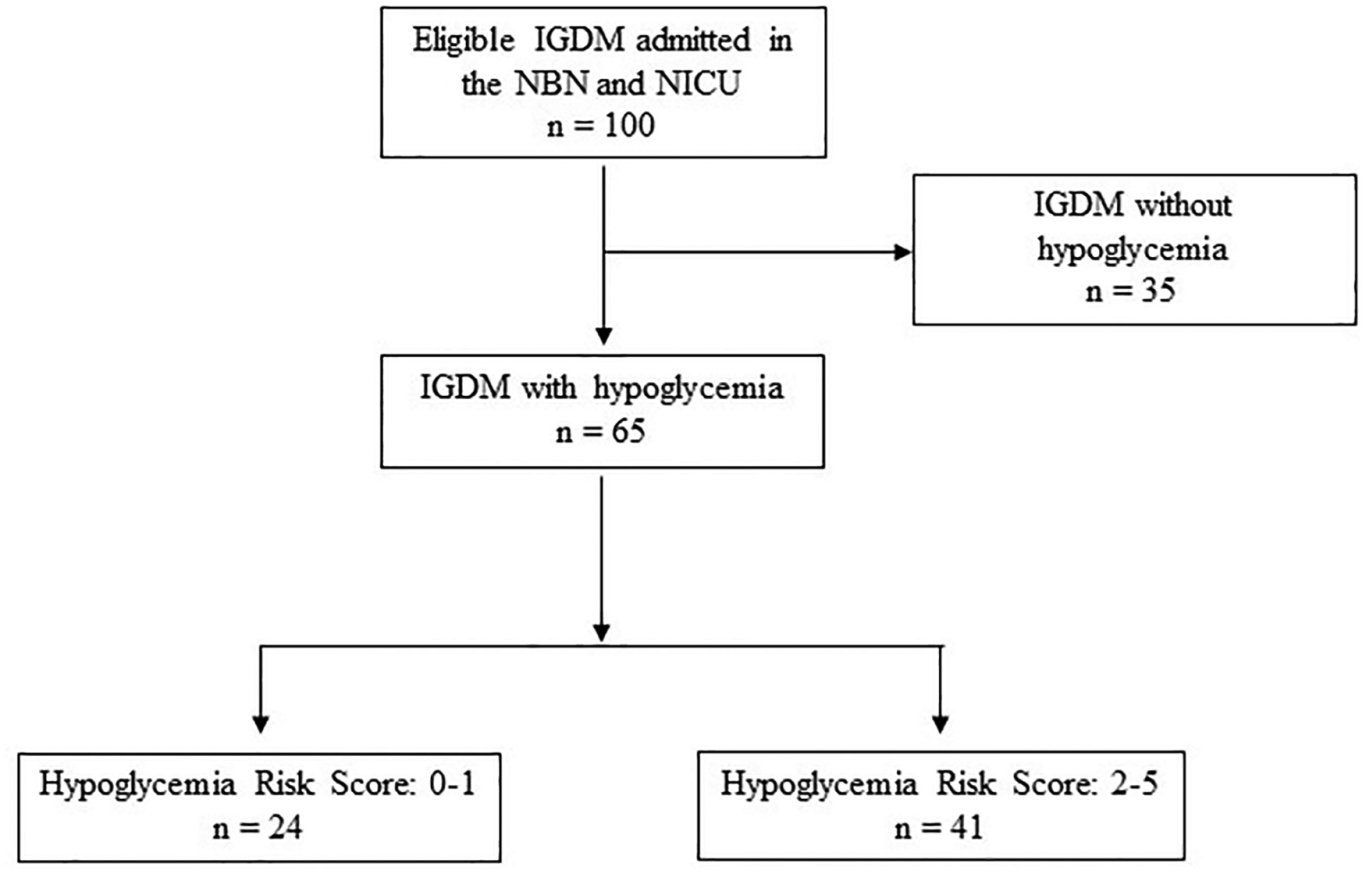
Flow diagram of study subjects.

We assessed 100 newborn IGDM for eligibility during the study period. Of the 100 infants, we excluded 35 infants because they did not have hypoglycemia. We, therefore, included 65 participants in the study. Twenty-four infants had a low HRS, and forty-one infants had a high HRS. The flow chart of all the IGDM reviewed during the study period is displayed in Figure 1.

### Demographics

The diagnosis of NH was made in 65 IGDM admitted during the study period. Of the 65 infants, 33 were females. Seven infants were Asian, 4 were black, 17 were Hispanic, and 37 were Caucasian. Using Fenton's growth curve (12), 77% were appropriate for gestational age, 12% were large, and 11% were small. Cesarean section was the method of delivery in 62% of the infants.

Of the 65 mothers with gestational diabetes, 68% were <35 years of age at delivery, 71% had a BMI > 30 Kg/m^2^, and 52% had drug-controlled gestational diabetes (GDMA2).

After evaluating baseline characteristics, we found no significant effect of gender, gestational age, race, size, maternal age, maternal BMI, or type of maternal gestational diabetes in the HRS groups. However, delivery by cesarean section was significantly higher in infants with high HRS than in infants with low HRS (*p* = 0.04). All the demographics is presented in Table 2.

**Table 2:** Demographic characteristics of study infants and mothers (*n* = 65).

Variable	Level	N	Score 0-1	Score 2-5	*P* value
Gender	F	33	36%	64%	0.92
	M	32	38%	62%	
Gestational age (Mean ± SD)	≥35weeks (wks.)	65	38.2 ± 1	38.0 ± 2	0.4
Race	Asian	7	29%	71%	0.68
	Black	4	25%	75%	
	Hispanic	17	29%	71%	
	Caucasian	37	43%	57%	
Size for dates	AGA	50	38%	62%	0.62
	LGA	8	25%	75%	
	SGA	7	43%	57%	
Delivery mode	Vaginal	25	52%	48%	0.04
	C-section	40	28%	73%	
Maternal age (Mean ± SD)	<35 y	44	28.4 ± 3.6	28.8 ± 4.2	0.72
	≥35 y	21	41.3 ± 3.9	38.2 ± 2.1	0.09
Maternal BMI (Mean ± SD)	<30 Kg/m^2^	19	27.3 ± 1.8	27.6 ± 1.8	0.76
	>30 Kg/m^2^	46	36.5 ± 8.5	38 ± 7.4	0.57
Maternal diabetic control	Diet-controlled	31	39%	50%	0.36
	Drug-controlled	34	61%	50%	

### Multiple logistic regression analysis

We found a relationship between gender and maternal pre-delivery glucose level on the HRS as seen in Table 3. Females are 5 times more likely to have an HRS ≥2 compared to males. In addition, each maternal pre-delivery glucose level increase by 1 mg/dl increases the likelihood of scoring ≥2 in HRS by 6%. However, the relationship between the mode of delivery and HRS is not statistically significant (*p* = 0.06).

**Table 3:** The influence of the independent variable on the hypoglycemia risk.

Factors	Odd Ratio	95% Confidence Interval		*P* value
Gestational age (weeks)	0.776	0.421	1.432	0.417
Gender (Female vs. male)	5.094	1.147	22.611	0.032
Birth weight (g)	1.000	0.999	1.002	0.618
Mode of delivery (C-section vs. vaginal)	3.653	0.953	14.004	0.059
Maternal age	1.081	0.955	1.224	0.220
Maternal pre-delivery glucose	1.062	1.009	1.118	0.021
Maternal BMI	0.948	0.878	1.024	0.173

A multiple logistic regression of the relationship between the Hypoglycemia Risk Scores (HRS) and the variables showed that females have 5.10 times higher likelihood to have a HRS ≥2 than males (95% CI 1.15–22.61, *p* = 0.03), and 1 mg/dl increase in maternal pre-delivery glucose will increase the likelihood of HRS ≥2 by 6% (95% CI 1.01–1.12, *p* = 0.02).

### Duration of hypoglycemia and treatment with or without intravenous dextrose

Of the 65 hypoglycemic IGDM included in the study, 23 (35%) required intravenous dextrose. More infants in the group who scored 2–5 on HRS required intravenous dextrose than those who scored 0–1 (20 vs. 3; *p* = 0.03).

Three IGDM that scored 0–1 on the HRS required intravenous dextrose: two infants had respiratory distress after 1 h of life, and one had persistent low glucose in the NBN. As a result, all three infants were transferred to the NICU. Almost all IGDM who continued on enteral feeds and scored 0–1 on HRS had resolution of their hypoglycemia by 3 h of age (95%). However, hypoglycemia persisted 3-hours after delivery in 10 infants (24.4%) who scored ≥2 on HRS. The duration of hypoglycemia and treatment are depicted in Table 4.

**Table 4:** Intravenous dextrose requirement and glucose resolution at 3 h of life in relation to HRS.

Hypoglycemia Risk Score (HRS)	IV Dextrose infusion (IDI)	No IV Dextrose infusion	Total	Number of infants with POC glucose ≥2.78 mmol/L by 3 HOL
Score 0–1	3^a^	21	24	21
Score 2–5	20	21	41	31
Total	23	42	65	52

12.5% of infants that scored 0-1 required intravenous dextrose, and was significantly different from intravenous dextrose requirement by infants that scored 2-5 (*p* = 0.03).

^a^ 2 out of the 3 had respiratory distress. IV, intravenous; HOL, hours of life; POC, point-of-care.

### Point of care glucose vs. plasma glucose levels

**Figure 2:**
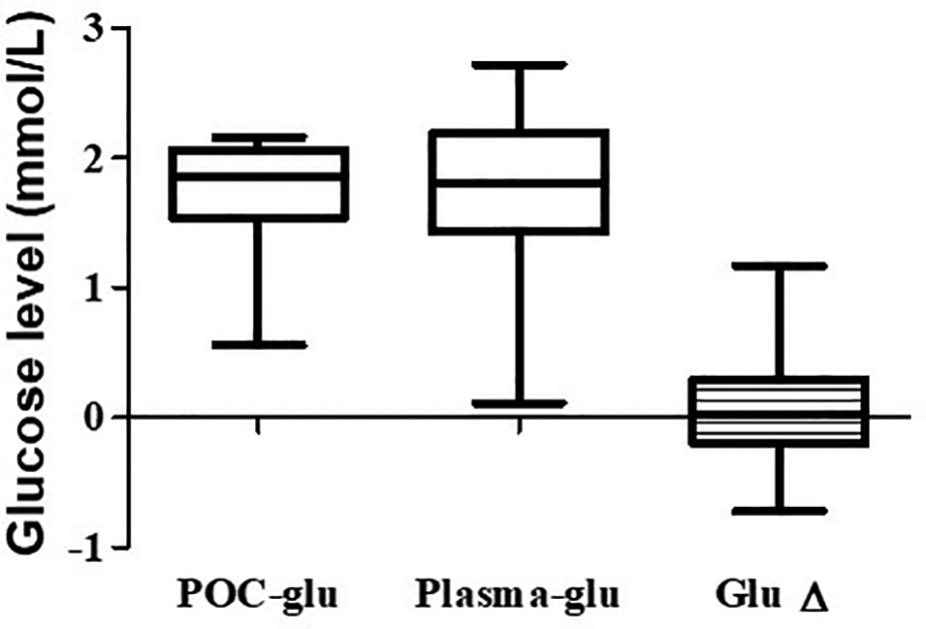
Comparison of POC and the plasma glucose levels. The median, first, and third quartiles are shown in the graph. The median difference between the POC and the plasma glucose is 0.05 mmol/L. POC, Point-of-care.

Thirty of sixty-five subjects (46%) had their blood redrawn for laboratory plasma glucose levels within 1–2 min of POC glucose levels <40 mg/dl (2.22 mmol/L). We found the median paired difference between the POC glucose and the plasma glucose was 0.05 mmol/L (interquartile ranges, 1.51–2.07; 1.43–2.19 mmol/L respectively, *p* = 0.81), as shown in Figure 2. Nevertheless, 20% (6 of 30) of the subjects with POC glucose <40 mg/dl (2.22 mmol/L) had plasma glucose levels >40 mg/dl (2.22 mmol/L) but <47 mg/dl (2.61 mmol/L), and another 6.7% (2 of 30) had a plasma glucose level of ≥47 mg/dl (2.61 mmol/L).

## Discussion

In a retrospective study of 65 hypoglycemic IGDMs born at ≥35 weeks gestational age, we found a strong association between low HRS at an hour of life and resolution of hypoglycemia without intravenous dextrose. In contrast, half of the neonates with high HRS required intravenous dextrose to resolve their hypoglycemia. These findings suggest that low HRS within an hour of life in IGDM may help identify infants who stay with their mothers and feed in the newborn nursery.

Although there was no significant effect of gender, gestational age, race, size, maternal age, maternal BMI, or type of maternal gestational diabetes on the HRS, we found in a univariate analysis that more infants with high HRS were born by C-section (*p* = 0.04). Notably, infants with high HRS were more likely to require intravenous dextrose for hypoglycemia treatment than infants with low HRS. This finding is consistent with the report by Turner et al. (13), who found that infants born *via* C-section had higher odds of requiring intravenous dextrose to treat NH when compared with infants born vaginally. Nevertheless, the relationship between C-sections and increased odds of intravenous dextrose for NH has yet to be thoroughly evaluated. This finding therefore, underscores the need for future studies to understand the factors that connect C-sections to intravenous dextrose requirements.

Our result showed that females in a multiple logistic regression have approximately 5% higher likelihood of scoring ≥2 in HRS than males (95% CI 1.15–22.61, *p* = 0.03). Although the reason for this relationship is unclear, females may be more sensitive to in-utero exposure to increased glucose levels than males (14, 15). Our observation is consistent with Ahel et al., who found in a recent study that the chances of developing hypoglycemia are increased three times by females compared to males gender (16).

Our observation that almost all IGDM who tolerated enteral feed and scored 0–1 on HRS had resolution of their hypoglycemia by 3 h of age was consistent with what was described by Agrawal et al. (17), who reported no hypoglycemia in IGDM after 2 h of life. Therefore, these findings suggest that frequent blood glucose monitoring beyond 3 h of life in IGDM who scored low on HRS may not be necessary.

In infants whose POC glucose levels were <40 mg/dl (2.22 mmol/L), we found no statistical difference between the POC and laboratory plasma glucose levels (*p* = 0.81). Our findings agree with Nuntnarumit et al., who showed no statistical difference between the Stat strip® POC glucose and plasma glucose levels (18). However, the Stat strip® POC glucose underestimated the glucose levels in 26.7% (8 of 30) of the patients. Our study, therefore, underscores the importance of confirming POC glucose <40 mg/dl (2.22 mmol/L) with laboratory plasma glucose.

We recognize various limitations and pitfalls with our present study. Thus, primary caregivers should consider these limitations when applying the findings to clinical practice. First, the research is limited to hypoglycemia in infants of gestational diabetic mothers. Therefore, we did not account for other conditions that increase the risk of hypoglycemia in newborns. We believe future studies should include large for gestational age infants regardless of maternal diabetic status.

The retrospective nature of this study represents a second limitation. There may be inaccuracies and data loss from the perinatal database completed by clinical staff. However, the glucose monitoring and assessment protocol are per the AAP's recommendation.

The use of a capillary sample for glucose monitoring is the third limitation. Glucose values obtained from capillary sampling can be unreliable if peripheral blood flow is not free-flowing (19). In addition, elevated hematocrit levels seen in neonates can interfere with blood glucose values (20). However, POC glucose using Stat strip® has a narrow margin of error and is not affected by hematocrit levels (18).

Finally, we conducted this single-centered study before our institution's routine use of glucose gel in managing early hypoglycemia. Although Weston PJ et al. (21) showed that dextrose gel did not alter the need for intravenous dextrose in treating hypoglycemia, our findings may still not apply to hypoglycemic IGDM receiving glucose gel. Therefore, a prospective multicenter study is required to assess the impact of oral dextrose gel on the HRS.

## Conclusion

Glucose screening is clinically crucial in infants of gestational diabetic mothers for timely recognition and treatment of hypoglycemia. However, early identifying IGDM whose hypoglycemia will or will not require intravenous dextrose for resolution is essential. Our results suggest that HRS discriminated between IGDM requiring intravenous dextrose and those not requiring intravenous dextrose to resolve hypoglycemia. The implementation of HRS, if replicated in a multicenter randomized trial, may facilitate early recognition of IGDM that will be triaged to either a newborn nursery for enteral feeds only or transfer for more invasive care.

## Data Availability

The raw data supporting the conclusions of this article will be made available by the authors, without undue reservation.

## References

[1] Robert MK Stanton BF St. Geme JW Schor NF Behrman RE. Infant of diabetic mothers. In: Carlo WA, editors. Nelson Textbook of pediatrics. 19th ed. Philadelphia: Elsevier (Saunders) (2012). p. 627–9.

[2] Nold JL Georgieff MK. Infant of diabetic mothers. Pedaitr Clin North Am. (2004) 51(3):619–37. 10.1016/j.pcl.2004.01.003 15157588

[3] Ward PM Deshpande S. Metabolic adaptation at birth. Semin Fetal Neonatal Med. (2005) 10(4):341–50. 10.1016/j.siny.2005.04.001 15916931

[4] Boluyt N van Kempen A Offringa M. Neurodevelopment after neonatal hypoglycemia: a systemic review and design of an optimal future study. Pediatrics. (2006) 117(6):2231–43. 10.1542/peds.2005-1919 16740869

[5] Burns CM Rutherford MA Boardman JP Cowan FM. Patterns of cerebral injury and neurodevelopmental outcomes after symptomatic neonatal hypoglycemia. Pediatrics. (2008) 122(1):65–74. 10.1542/peds.2007-2822 18595988

[6] Dalgic N Ergenekon E Soysal S Koc E Atalay Y Gucuyener K. Transient neonatal hypoglycemia- long term effects on neurodevelopmental outcome. J Pediatr Endocrinol Metab. (2002) 15(3):319–24. 10.1515/JPEM.2002.15.3.319 11924935

[7] Kinnala A Rikalainen H Lapinleimu H Parkkola R Kormano M Kero P. Cerebral magnetic resonance imaging and ultrasonography findings after neonatal hypoglycemia. Pediatrics. (1999) 103(4 Pt 1):724–29. 10.1542/peds.103.4.724 10103293

[8] Adamkin DH. Committee on fetus and newborn. Postnatal glucose homeostasis in late preterm and term infants. Pediatrics. (2011) 127(3):575–79. 10.1542/peds.2010-3851 21357346

[9] Scheurer-Monaghan A Stevens T Haider-Ahmad Z Lowmaster-Csont GA Guillet R. Delivery room triage of infants of medication dependent diabetic mothers (IMDDM): validation of a risk score for hypoglycemia. (abstract, poster 23). 21st Annual meeting, eastern society for pediatric research; 2009, March 13-15; Philadelphia, PA

[10] Ramos GA Hanley AA Aguayo J Warshak CR Kim JH Moore TR. Neonatal chemical hypoglycemia in newborns from pregnancies complicated by type 2 and gestational diabetes mellitus- the importance of neonatal ponderal index. J Matern Fetal Neonatal Med. (2012) 25(3):267–71. 10.3109/14767058.2011.573828 21557689

[11] Lucas A Morley R Cole TJ. Adverse neurological outcome of moderate neonatal hypoglycemia. Br Med J. (1988) 297(6659):1304–08. 10.1136/bmj.297.6659.1304 2462455 PMC1834933

[12] Fenton TR Kim JH. A systemic review and meta-analysis to revise the fenton growth chart for preterm infants. BMC Pediatr. (2013) 13:59. 10.1186/1471-2431-13-59 23601190 PMC3637477

[13] Turner D Monthé-Drèze C Cherkerzian S Gregory K Sen S. Maternal obesity and cesarean section delivery: additional risk factors for neonatal hypoglycemia? J Perinatol. (2019) 39(8):1057–64. 10.1038/s41372-019-0404-z 31213637 PMC6660417

[14] Krishnaveni GV Veena SR Hill JC Kehoe S Karat SC Fall CH. Intrauterine exposure to maternal diabetes is associated with higher adiposity and insulin resistance and clustering of cardiovascular risk markers in Indian children. Diabetes Care. (2010) 33:402–4. 10.2337/dc09-1393 19918007 PMC2809291

[15] Gerlini G Arachi S Gori MG Gloria F Bonci E Pachi A. Developmental aspects of the offspring of diabetic mothers. Acta Endocrinol. (1986) 277:150–5. 10.1530/acta.0.111S0150 3464148

[16] Butorac Ahel I Lah Tomulić K Vlašić Cicvarić I Žuvić M Baraba Dekanić K Šegulja S Incidence and risk factors for glucose disturbances in premature infants. Medicina (Kaunas). (2022) 58(9):1295. 10.3390/medicina58091295 36143971 PMC9501184

[17] Agrawal RK Lui K Gupta JM. Neonatal hypoglycemia in infants of diabetic mothers. J Paediatr Child Health. (2000) 36(4):354–6. 10.1046/j.1440-1754.2000.00512.x 10940170

[18] Nuntnarumit P Chittamma A Pongmee P Tangnoo A Goonthon S. Clinical performance of the new glucometer in the nursery and neonatal intensive care unit. Pediatr Int. (2011) 53(2):218–23. 10.1111/j.1442-200X.2010.03214.x 21501305

[19] Williams AF. Hypoglycemia of the newborn: a review. Bull World Health Organ. (1997) 75(3):261–90. PMID: ; PMCID: 9277014 PMC2486945

[20] Aynsley-Green A. Glucose: a fuel for thought!. J Paediatr Child Health. (1991) 27(1):21–30. 10.1111/j.1440-1754.1991.tb00340.x 1828360

[21] Weston PJ Harris DL Battin M Brown J Hegarty JE Harding JE. Oral dextrose gel for the treatment of hypoglycemia in newborn infants. Cochrane Database Syst Rev. (2016)(5):CD011027. 10.1002/14651858.CD011027.pub2 27142842

